# Is postpartum depression related to total weight gain during pregnancy and maternal anemia?

**DOI:** 10.1590/1806-9282.20230908

**Published:** 2024-03-04

**Authors:** Serap Topkara Sucu, Elif Karaman, Caner Kose, Sadun Sucu, Hüseyin Levent Keskin

**Affiliations:** 1 Ankara Etlik City Hospital, Department of Gynecology and Obstetrics – Ankara, Turkey.; 2 Ankara Etlik City Hospital, Department of Psychology – Ankara, Turkey.; 3 Ankara Etlik City Hospital, Department of Perinatology – Ankara, Turkey.

**Keywords:** Anemia, Depression, postpartum

## Abstract

**OBJECTIVE:**

This study aimed to investigate the effects of weight gain and maternal anemia on postpartum depression.

**METHODS:**

This is a prospective, single-center, case-control study. We recorded the demographic characteristics, blood ferritin level, and weight gain during the pregnancy. This study was planned between April 2023 and June 2023 in the Obstetrics and Gynecology Clinic of Ankara Etlik City Hospital. A total of 109 patients were enrolled in the study. Patients were assessed with the Edinburgh Postpartum Depression Scale. Weight gain, nutritional education, educational level, mode of delivery, and pregnancy history were asked in person. Ferritin levels at the onset of labor were determined to detect anemia. Twin births, births due to fetal anomalies or intrauterine stillbirths, patients with systemic infections, and patients diagnosed with a psychiatric disorder in the past 6 months whose records were not accessible were excluded from the study.

**RESULTS:**

Pregnancy weight gain and percentage of pregnancy weight gain were higher. Serum ferritin levels and nutritional education during pregnancy were lower in the postpartum depression group (p<0.001). These parameters with statistical significance were identified as risk factors in the regression analysis for postpartum depression (p<0.05). In receiver operating characteristics analysis, >15 kg for weight gain, >28.8 for percentage of weight gain in pregnancy, and <19 ng/dL for serum ferritin level were identified as cutoff values (p<0.001).

**CONCLUSION:**

Nutritional education and vitamin supplementation should be recommended to pregnant women during routine examinations.

## INTRODUCTION

Pregnancy is an important period in which physiological, psychological, and social changes take place in women’s lives which must be adapted. During this period, the psychosocial factors and hormonal changes are a high risk of developing depression^1^. Postpartum depression (PPD) is common and is estimated to occur in one in five women after childbirth^2^. Both the physiological alterations occurring during pregnancy and the postpartum responsibility for demands of infant care, alongside conditions such as insomnia, fatigue, and a lack of personal time, may contribute to maternal distress and the development of PPD. In addition, if it occurs during puerperium, it can have negative consequences on the child’s growth and development. Therefore, special attention should be paid to the diagnosis and early treatment of PPD^3^. Studies on physiological changes during pregnancy show that 8–12% of women suffer from problems such as generalized anxiety and obsessive-compulsive disorder in the postpartum period, and these problems can reach up to 19% depression^4,5^. As emotional disorders, including depression, exert a detrimental influence on a woman’s physical and psychological health, they can also lead to her inability to adequately perform her parental and caregiving duties^6^.

Anemia is one of the most common diseases during pregnancy. More than 40% of pregnant women in the world suffer from anemia, according to the World Health Organization (WHO). Anemia during pregnancy or in the postpartum period has been identified as a possible physiological risk factor for PPD, as anemia can lead to fatigue, irritability, apathy, and depressive symptoms^7^.

An important factor in physical changes is excessive weight gain during pregnancy. Even if there is no weight problem before pregnancy, excessive weight gain during pregnancy alone poses a risk for pregnancy complications^8^. Obesity before pregnancy, excessive weight gain during pregnancy, and weight gain after delivery are observed in approximately 50% of women^9^. Excessive weight gain during pregnancy leads to self-care problems and increases the propensity for PPD^10^.

In this study, we aimed to reveal the effects of anemia and weight gain in pregnancy on PPD.

## METHODS

### Participants

This study was planned between April 2023 and June 2023 in the Obstetrics and Gynecology Clinic of Ankara Etlik City Hospital as an analytical case-control study on 109 patients who gave birth and applied for postpartum outpatient examination.

The inclusion criteria were as follows: patients who gave birth in our institution, who have similar characteristics in terms of number of births and age of births so as not to disturb the integrity of the sample, and women who applied after birth were included in the study.

The exclusion criteria were as follows: Twin births, births due to fetal anomalies or intrauterine stillbirths, patients with systemic infections, and patients who had been diagnosed with a psychiatric disorder in the previous 6 months and whose records could not be accessed were excluded from the study.

#### Edinburgh Postpartum Depression Scale

Edinburgh Postpartum Depression Scale (EPDS) was developed by Cox et al., and its Turkish validity and reliability study was performed by Aydın Nazan et al.^11,12^. The EPDS is used to determine the risk of PPD and to measure the change in severity. The scale consists of 10 items, and each item is evaluated according to a 4-point Likert-type scale in the range of 0–3. The minimum score to be obtained from the scale is 0, whereas the maximum score is 30, and individuals who have a score of higher than 12 are evaluated as high risk in terms of depression. The instructions are explained to the people and they are asked to tick the statement that most corresponds to them. As a result of the study conducted in Turkey, the cutoff value of the scale was calculated as 12. Patients with an Edinburgh score of 12 or more were classified as PPD and referred to a psychologist. Patients with an EPDS score below 12 were considered control group 1, and patients with an EPDS score above 12 were considered group 2. The parameters were studied and compared between these two groups.

Nutrition education: Patients participating in the study were given initial nutrition education at the prenatal pregnancy school. Patients who wanted more were referred to a dietitian regularly in each trimester, and a separate diet program was created for each patient regarding the calorie intake they should consume.

A 16-parameter questionnaire was used to follow up cases, and patient demographic, laboratory, and postpartum Edinburgh values were recorded. The patients’ prenatal and postnatal hemoglobin levels, ferritin levels at the beginning of pregnancy, and weight gain during pregnancy were obtained from the patients’ records. These parameters were compared between the patient and control groups.

This study was approved by the Ethics Committee for Noninterventional Clinical Research of Ankara Etlik City Hospital and conducted in accordance with the ethical standards of the 1964 Declaration of Helsinki and subsequent amendments (approval no. AESH-EK1-2023/164, dated May 17, 2023).

Informed consent was obtained from all participants.

### Statistical analysis

All statistical analyses were performed using RStudio for statistical computing to analyze the data. The variables were investigated using the Shapiro-Wilk test to determine whether or not they are normally distributed. Descriptive analyses were expressed as mean and standard deviation for parametric data, median and quartiles for nonparametric data, and frequencies and percentages for categorical data. The independent-samples t-test was used for parametric data, the Mann-Whitney U test for nonparametric data, and the chi-square test for categorical data. The capacity of various parameters that can be used to predict PPD was analyzed using the receiver operating characteristics (ROC) curve analysis. For the multivariate analysis, the possible factors identified with univariate analyses were further entered into the binary logistic regression analysis to determine additional independent predictors of PPD. A p<0.05 was considered statistically significant.

## RESULTS

A total of 150 participants were identified from the initial submissions. After applying the inclusion and exclusion criteria, 15 (10%) patients from the earthquake area, 12 (8%) patients taking psychotropic drugs, 5 (3%) patients with fetal anomalies, and 9 (6%) patients who had delivered prematurely due to pregnancy complications were excluded. Thus, a total of 109 participants were eligible for this study. Patients were divided into two groups based on Edinburgh scores. Patient demographic characteristics, parameters compared, and statistically significant and nonsignificant results along with p-value are shown in Table 1.

**Table 1.** Baseline demographic and clinical characteristics of the study population.

Variables		Group I (control) (n=58)	Group II (depression group) (n=51)	Total (n=109)	p
Age (years)		28.8±5.28	27.5±5.03	28.2±5.18	0.203
Gravida (number)		2 (1–3)	2 (1–3)	2 (1–3)	0.316
Parity (number)		2 (1–3)	1 (1–2)	2 (1–2)	**0.049**
Miscarriage (number)		0 (0–1)	0 (0–1)	0 (0–1)	0.382
Maternal weight (kg) (at the beginning of pregnancy)		64 (59.8–72.5)	65 (55–80)	64 (57–75.5)	0.862
Maternal weight (kg) (at the end of pregnancy)		76.5 (70–85)	81 (70–99)	78 (70–86.5)	0.069
BMI (kg/m^2^) (at the beginning of pregnancy)		25.1 (22.7–27.9)	24 (20.3–30.5)	24.5 (21.9-28.4)	0.502
BMI (kg/m^2^) (at the end of pregnancy)		29 (26.8–31.3)	30 (26–35)	29 (26–33)	0.363
Weight gained during pregnancy (kg)		10 (8.8–14)	16 (12–20)	13 (10–16)	**<0.001**
Percentage of weight gained during pregnancy (%)		15.9 (12.6–22.1)	22.7 (15.6–31.7)	18.5 (13.3–25)	**<0.001**
Serum ferritin level (ng/dL) (at the beginning of pregnancy)		22.5 (14.5–31.3)	7.2 (5–14)	15 (7–25)	**<0.001**
Serum hemoglobin levels (g/dL) (at the end of pregnancy)		10.5 (10–11.2)	10.6 (9.3–12)	10.5 (9.6–11.4)	0.983
Serum hemoglobin levels (g/dL) (at the beginning of pregnancy)		12.1 (11.6–12.9)	12.3 (11.3–13.5)	12.1 (11.6–11.3)	0.541
Edinburg score		4 (3-6)	16 (14–18)	8 (4–16)	**<0.001**
Type of delivery	nvd	20 (34.5)	18 (35.3)	38 (34.9)	1.000
	cs	38 (65.5)	33 (64.7)	71 (65.1)	
Nutrition education during pregnancy	Yes	4 (6.9)	43 (84.3)	47 (43.1)	**<0.001**
	No	54 (93.1)	8 (15.7)	62 (56.9)	
Educational status					
High school graduation		35 (60.3)	42 (82.4)	77 (70.6)	**0.021**
Bachelor’s degree		23 (39.7)	9 (17.6)	32 (29.4)	

nvd: normal vaginal delivery; cs: C-section. Data are expressed as mean±standard deviation, median (Q1–Q3), or number (percentage,) where appropriate. A p<0.05 indicates a significant difference. Statistically significant p-value are denoted in bold.

There is an association between weight gain during pregnancy and PPD. An analysis of ROC showed that PPD can occur when weight gain during pregnancy exceeds 15 kg. The ferritin level at the beginning of pregnancy is statistically significantly lower in group 2 compared with group 1 (p<0.001). In the ROC analysis performed after it was found that ferritin at the beginning of pregnancy is also associated with PPD, PPD can be monitored at a serum ferritin level of 19 (ng/dL) and below, which indicates psychopathology of the patient (p<0.001). Similarly, PPD can occur when the weight gain is 28.8% or more compared with the patient’s baseline weight (Table 2).

**Table 2.** Receiver operating characteristics curve analysis results of parameters that can be used to predict postpartum depression.

Variables	AUC (95%CI)	P	Cutoff value	Sensitivity (%)	Specificity (%)	(+) LHR	(–) LHR
Weight gained during pregnancy (kg)	0.743 (0.650–0.822)	**<0.001**	>15	50.9	91.3	5.91	0.54
Percentage of weight gained during pregnancy (%)	0.695 (0.600–0.780)	**<0.001**	>28.8	37.2	98.2	21.61	0.64
Serum ferritin level (ng/dL) (at the beginning of pregnancy)	0.840 (0.757–0.903)	**<0.001**	≤19	88.2	68.9	2.84	0.17

AUC: area under the curve; LHR: likelihood ratio. A p<0.05 indicates a significant difference. Statistically significant p-value are denoted in bold.

The regression analysis performed showed that each 1 kg increase in maternal weight during pregnancy increased the risk for PPD by 1.206-fold (p=0.036). Each 1% increase in weight compared with baseline weight during pregnancy increased the risk for PPD by 1.104-fold (p=0.022), and lack of nutritional education during pregnancy increased the risk for PPD by 46.02-fold (p<0.001). Each 1 ng/dL increase in ferritin early in pregnancy resulted in 1.12-fold protection from PPD (Table 3).

**Table 3.** Binary logistic regression results and risk coefficients of parameters for postpartum depression.

Variables	OR (95%CI)	p
Weight gained during pregnancy (kg)	1.206 (1.013–1.436)	**0.036**
Percentage of weight gained during pregnancy (%)	1.104 (1.014–1.202)	**0.022**
Serum ferritin level (ng/dL) (at the beginning of pregnancy)	0.886 (0.822–0.955)	**0.002**
Lack of nutrition education during pregnancy	46.02 (10.61–199.66)	**<0.001**
Gravida	1.066 (0.639–1.780)	0.806
Parity	0.413 (0.070–2.440)	0.351

A p<0.05 indicates a significant difference. Statistically significant p-value are denoted in bold.

## DISCUSSION

In this study, the relationship between PPD and weight gain during pregnancy, nutritional education, and ferritin levels was investigated. It was found that weight gain during pregnancy and the percentage of weight gain were significantly higher in the group with PPD than in the healthy group. These results suggest that weight gain, nutrition during pregnancy, and low ferritin levels (iron deficiency) may increase the risk of PPD.

The conditions that influence PPD have been studied in the literature^13,14^. A study conducted by Eberhard-Gran et al., found that the prevalence of depression was higher in postpartum women than in non-postpartum women^15^. The study conducted by Oztora et al., found that the prevalence of PPD was 14% in the first month after delivery and 17% in the second month. The likelihood of PPD was significantly higher among mothers who were younger, had low income, were not breastfeeding, whose spouse was not working, and whose child had health problems. However, there was no significant between mothers’ educational level and PPD^16^. Although there was no significant difference between educational levels, there were more college graduates in the healthy group. This could indicate that education level influences dietary behavior. The study found that nutrition education was significantly higher in the healthy group, which was likely related to educational status.

We found the participation rate in nutrition education was statistically low in the group with PPD. This result suggests that nutrition education during pregnancy has a positive effect on reducing the risk of PPD.

Anemia during pregnancy is a major problem in both developed and developing countries. The most common cause of anemia is iron deficiency, and evidence suggests that up to 90% of anemia in mothers is due to inadequate dietary iron intake^17^. In our study, serum ferritin levels at the beginning of pregnancy were examined as a marker for iron deficiency anemia. Ferritin levels during pregnancy were associated with PPD in agreement with the literature^18^. Low ferritin level was associated with a higher risk of PPD. This finding suggests that ferritin level has the potential value as a biomarker for monitoring or predicting PPD.

Similar to the literature, we showed that weight gain during pregnancy increases the risk of PPD. In the study by Fraga et al., 32% of women participating in the study were obese, whereas the rate of PPD was 26.3%. Compared with women who were obese before pregnancy and with normal-weight women, the risk of developing PPD was higher in women with obesity^19^. A study conducted by Zanardo et al., examined the effect of weight gain during pregnancy (PPD), and the results were different from our study. Excessive weight gain during pregnancy was found in 388 (30.6%) of the 1,268 women involved in the study. However, no association was found between EPDS scores and weight gain^20^. In a study in which Fışkın and colleagues examined the association between visual and physical changes during pregnancy and psychological complaints in the postpartum period, 59% of women were found to be overweight in the postpartum period. Postpartum women are satisfied with their body, and there is a weak negative relationship between it and their psyche^21^.

This research has some limitations. The pregnant women were selected from a single institution. Our hospital is a newly opened facility, our sample size is small, and therefore our results are not fully representative of the population. Another limitation is that a single survey evaluation was made to the patients, and no re-evaluation was made after the psychologist’s examination.

## CONCLUSION

This study showed that weight gain during pregnancy, nutritional education, and ferritin levels have been shown to influence the risk of PPD. In light of these findings, these factors should be considered in prenatal care. The importance of prenatal care should be explained to the whole society. There is a need for prospective longitudinal studies that fully evaluate the population, including a larger number of patients, in the form of multicenter studies with different institutions.

## References

[1] Learman LA. (2018). Screening for depression in pregnancy and the postpartum period.. Clin Obstet Gynecol..

[2] Wisner KL, Sit DK, McShea MC, Rizzo DM, Zoretich RA, Hughes CL (2013). Onset timing, thoughts of self-harm, and diagnoses in postpartum women with screen-positive depression findings.. JAMA Psychiatry..

[3] Field T. (2010). Postpartum depression effects on early interactions, parenting, and safety practices: a review.. Infant Behav Dev..

[4] O’Hara MW, McCabe JE. (2013). Postpartum depression: current status and future directions.. Annu Rev Clin Psychol..

[5] Bolton JL, Wiley MG, Ryan B, Truong S, Strait M, Baker DC (2017). Perinatal western-type diet and associated gestational weight gain alter postpartum maternal mood.. Brain Behav..

[6] Silveira ML, Ertel KA, Dole N, Chasan-Taber L. (2015). The role of body image in prenatal and postpartum depression: a critical review of the literature.. Arch Womens Ment Health..

[7] Murray-Kolb LE, Beard JL. (2009). Iron deficiency and child and maternal health.. Am J Clin Nutr..

[8] Goldstein RF, Abell SK, Ranasinha S, Misso M, Boyle JA, Black MH (2017). Association of gestational weight gain with maternal and infant outcomes: a systematic review and meta-analysis.. JAMA..

[9] Salmon C, Sauve RS, LeJour C, Fenton T, Metcalfe A. (2020). A single gestational weight gain recommendation is possible for all classes of pregnant women with obesity.. Obes Res Clin Pract..

[10] Faith MS, Butryn M, Wadden TA, Fabricatore A, Nguyen AM, Heymsfield SB. (2011). Evidence for prospective associations among depression and obesity in population-based studies.. Obes Rev..

[11] Cox JL, Holden JM, Sagovsky R. (1987). Detection of postnatal depression. Development of the 10-item Edinburgh Postnatal Depression Scale.. Br J Psychiatry..

[12] Aydin N, Inandi T, Yigit A, Hodoglugil NN. (2004). Validation of the Turkish version of the Edinburgh Postnatal Depression Scale among women within their first postpartum year.. Soc Psychiatry Psychiatr Epidemiol..

[13] Kahveci G, Kahveci B, Aslanhan H, Bucaktepe PGE. (2021). Evaluation of prevalence and risk factors for postpartum depression using the Edinburgh Postpartum Depression Scale: a cross-sectional analytic study.. Gynecol Obstet Reprod Med..

[14] Ertürk Aksakal S, Pay RE, Köse C, Özkan D, EngiN Üstün Y. (2022). The effect of hydrotherapy applied during the active phase of labor on postpartum depression: a case-control study.. J Clin Obstet Gynecol..

[15] Eberhard-Gran M, Eskild A, Tambs K, Samuelsen SO, Opjordsmoen S. (2002). Depression in postpartum and non-postpartum women: prevalence and risk factors.. Acta Psychiatr Scand..

[16] Oztora S, Arslan A, Caylan A, Dagdeviren HN. (2019). Postpartum depression and affecting factors in primary care.. Niger J Clin Pract..

[17] Appiah PK, Nkuah D, Bonchel DA. (2020). Knowledge of and adherence to anaemia prevention strategies among pregnant women attending antenatal care facilities in Juaboso district in western-north region, Ghana.. J Pregnancy..

[18] Wassef A, Nguyen QD, St-André M. (2019). Anaemia and depletion of iron stores as risk factors for postpartum depression: a literature review.. J Psychosom Obstet Gynaecol..

[19] Fraga ACSA, Theme-Filha MM. (2020). Pregestational overweight and obesity and symptoms of postpartum depression: data from the Birth in Brazil Study.. J Affect Disord..

[20] Zanardo V, Giliberti L, Giliberti E, Grassi A, Perin V, Parotto M (2021). The role of gestational weight gain disorders in symptoms of maternal postpartum depression.. Int J Gynaecol Obstet..

[21] Fişkin G, Işik C. (2022). The relationship of visual and bodily changes experienced during pregnancy with body liking and psychological complaints in the postpartum period.. Dokuz Eylul Univ Facult Nurs Electronic J..

